# EphA6 promotes angiogenesis and prostate cancer metastasis and is associated with human prostate cancer progression

**DOI:** 10.18632/oncotarget.4088

**Published:** 2015-05-27

**Authors:** Shibao Li, Yingyu Ma, Chongwei Xie, Zhiyuan Wu, Zhihua Kang, Zujun Fang, Bing Su, Ming Guan

**Affiliations:** ^1^ Department of Laboratory Medicine, Huashan Hospital, Shanghai Medical College, Fudan University, Shanghai 200040, China; ^2^ Department of Pharmacology and Therapeutics, Roswell Park Cancer Institute, Buffalo, NY 14263, USA; ^3^ Biomedical Research Institute, Shenzhen-PKU-HKUST Medical Center, Shenzhen, Guangdong 518036, China; ^4^ Department of Urology, Huashan Hospital, Shanghai Medical College, Fudan University, Shanghai 200040, China; ^5^ Xinxiang Key Lab of Translational Cancer Research, The Third Affiliated Hospital, Xinxiang Medical University, Xinxiang, Henan 453003, China; ^6^ Department of Epidemiology and Environmental Health, School of Public Health and Health Professions, The State University of New York, Buffalo, NY 14214, USA

**Keywords:** Eph, ephrin, EphA6, metastasis, prostate cancer

## Abstract

Metastasis is the primary cause of prostate cancer (CaP)-related death. We investigate the molecular, pathologic and clinical outcome associations of EphA6 expression and CaP metastasis. The expression profiling of Eph receptors (Ephs) and their ephrin ligands was performed in parental and metastatic CaP cell lines. Among Ephs and ephrins, only EphA6 is consistently overexpressed in metastatic CaP cells. Metastatic potential of EphA6 is assessed by RNAi in a CaP spontaneous metastasis mouse model. EphA6 knock-down in human PC-3M cells causes decreased invasion *in vitro* and reduced lung and lymph node metastasis *in vivo*. In addition, knock-down of EphA6 decreases tube formation *in vitro* and angiogenesis *in vivo*. EphA6 mRNA expression is higher in 112 CaP tumor samples compared with benign tissues from 58 benign prostate hyperplasia patients. Positive correlation was identified between EphA6 expression and vascular invasion, neural invasion, PSA level, and TNM staging in CaP cases. Further, genome-wide gene expression analysis in EphA6 knock-down cells identified a panel of differentially regulated genes including PIK3IPA, AKT1, and EIF5A2, which could contribute to EphA6-regulated cancer progression. These findings identify EphA6 as a potentially novel metastasis gene which positively correlates with CaP progression. EphA6 may be a therapeutic target in metastatic CaP.

## INTRODUCTION

Prostate cancer (CaP) remains the most diagnosed non-cutaneous cancer and the second leading cause of cancer death in American men [1]. Metastasis is the primary cause of CaP-related death [2]. The five-year relative survival rate for men diagnosed with distal metastatic CaP is 28%, while the survival rate for those with local or regional CaP is nearly 100% (American Cancer Society, www.cancer.org). The overall or CaP-specific survival has not changed in the last 20 years in men presented with metastatic CaP, although an almost 40% decrease in prostate cancer mortality has been observed over the last 25 years in the US. Therefore, how to prevent CaP progression and treat metastasis remains a major clinical challenge.

Eph receptors (Ephs) represent the largest tyrosine kinase receptor family with 14 members in mammals [3]. Their ephrin ligand family has 8 members [4]. Ephs family exerts complex activities in cancer, which may lead to intriguing paradoxical effects. Interestingly, many effects caused by Ephs are mediated by signaling cascades modulating the actin cytoskeleton, cell-cell and cell-substrate adhesion, intercellular junctions, cell movement, and neovascularization [5–7]. Compelling evidence indicates that Ephs and their ephrin ligands are involved in many processes of metastasis, including adhesion, migration, invasion, and angiogenesis [4], suggesting that these family members are potential therapeutic targets for cancer metastasis.

We previously demonstrated that normal prostate tissue expresses a high level of EphA7, which is progressively lost during CaP development [8]. In line with our findings, EphA7 is not expressed in the lymph node and bone metastasis of prostate cancer [9]. In this study, we sought to examine more comprehensively the expression profile of all currently known human Ephs and their ephrin ligands in well-established CaP cell lines (LNCaP and PC-3) as well as in their derivative lymph node metastatic cell lines by real-time quantitative RT-PCR (qRT-PCR). We observed that EphA6 was the only consistently overexpressed member in both lymph node metastatic cell lines. We further investigated the potential role of EphA6 in CaP cell invasion, angiogenesis, and metastasis *in vitro* and also in a xenograft CaP mouse model. In addition, we examined EphA6 mRNA expression in 112 CaP tumor tissue samples and 58 BPH tissue samples, and analyzed the potential association of EphA6 expression with clinical characteristics including age, PSA value, prostate volume, Gleason score, tumor stage, vascular invasion and neural invasion. Our findings indicate a role for EphA6 in CaP invasion and metastasis and provide a strong foundation for further evaluation of EphA6 as a therapeutic target in CaP metastasis.

## RESULTS

### Up-regulation of EphA6 mRNA and protein in lymph node metastasis of CaP cells

**Figure 1:**
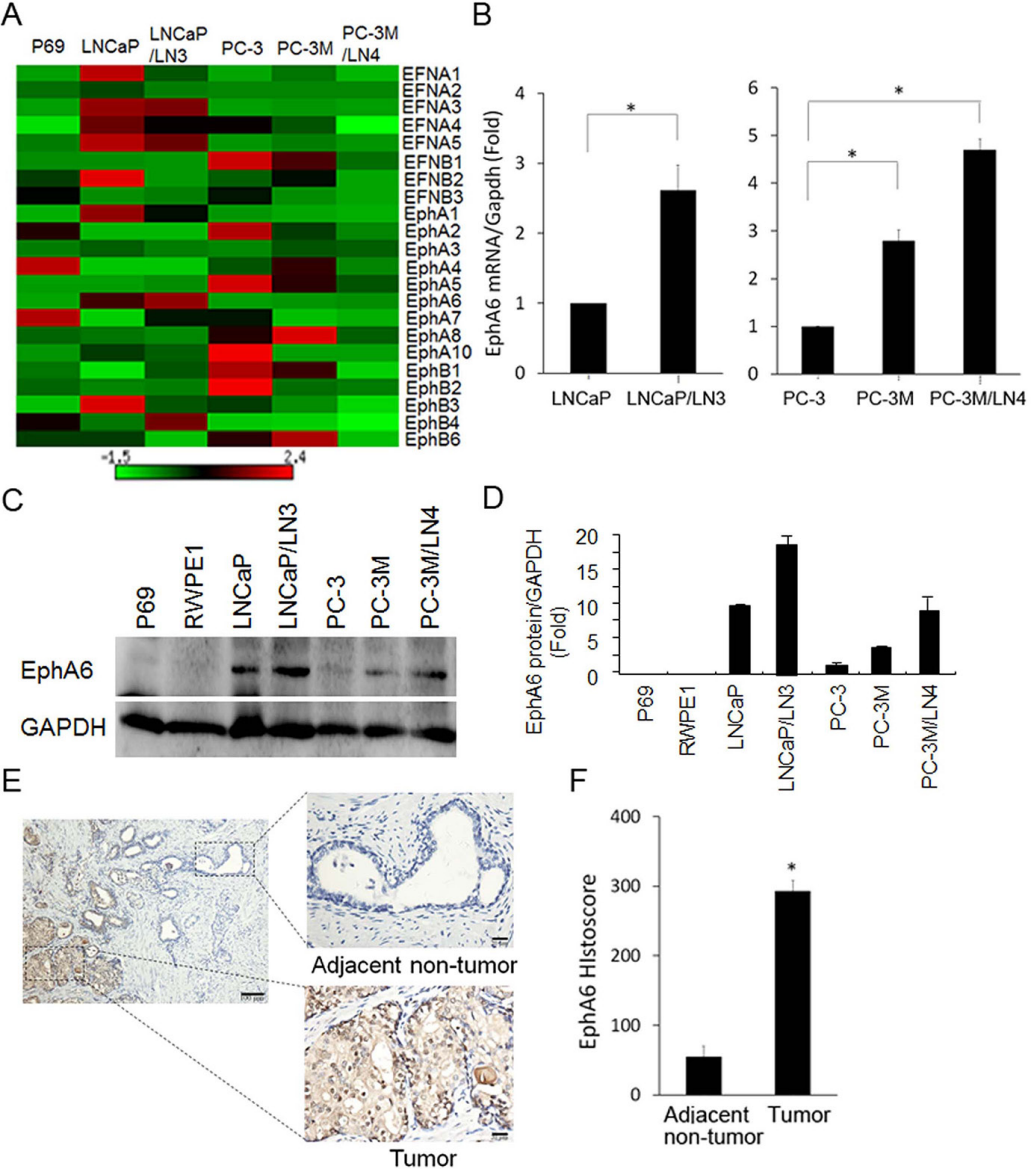
EphA6 mRNA and protein expression is up-regulated in CaP lymph node metastatic cell lines and CaP tumor tissues **A.** The mRNA expression of all currently known members of human Eph receptor and ephrins was detected and quantitated by qRT-PCR in CaP cell lines LNCaP, PC-3, PC-3M and their derivative lymph node metastatic lines LNCaP/LN3 and PC-3M/LN4. Immortalized prostate epithelial cell line P69 was used as a control cell line. GAPDH was used as a house keeping gene control. The heat-map depicts the mRNA expression of Eph and ephrin family members. **B.** The expression of EphA6 mRNA was also shown in bar graphs for clearer presentation. *P < 0.05. **C-D.** EphA6 protein expression in human CaP cell lines was examined by Western blot analysis (C), which was quantified by densitometry (D) Error bars, SE of three independent experiments. **E.** EphA6 protein expression was examined in primary human CaP tumor tissues and matched adjacent non-tumor tissues by IHC. Representative images of the IHC study of EphA6 expression in primary CaP tumor tissues and matched adjacent non-tumor tissues were presented. Magnifications: × 200 (left, bar, 100 μm) and × 400 (right, bar, 20 μm). **F.** The relative EphA6 histoscores were presented. *P < 0.05.

Ephs and their ligand ephrins are involved in the carcinogenesis of various human malignancies. The expression profiles of Ephs and ephrins has been characterized in a broad spectrum of human tumor tissues [10, 11]. However, the expression profile of the entire families of Ephs and ephrins is still unknown. In addition, the potential association between the expression of Eph families and metastasis remains unclear. Thus, we investigated the expression profiles of all currently known human Ephs and ephrins by qRT-PCR in CaP cell lines LNCaP, PC-3, metastatic PC-3M, and their lymph node metastatic cell lines LNCaP/LN3 and PC-3M/LN4. We observed that most members of the Ephs and ephrins family were expressed in all cell lines investigated, but the relative amounts of the transcripts varied considerably (Fig. 1A). This finding is consistent with a previous report evaluating Eph expression in breast cancer cell lines [10]. Although each cell line has a unique pattern of expression, we observed some remarkable patterns. Among Eph receptors, EphA6 expression is increased consistently and significantly in both lymph node metastasis derivative cell lines LNCaP/LN3 and PC-3M/LN4 compared with their parental cell lines (*P* < 0.01) (Fig. 1A). To more clearly show EphA6 mRNA expression in the LNCaP and PC-3M cell lines, EphA6 mRNA expression data was presented separately in bar graphs where EphA6 mRNA level in parental LNCaP or PC-3 cells was normalized to 1 (Fig. 1B). This novel finding suggests that EphA6 may be associated with CaP metastasis. To study the protein expression of EphA6, Western blot analysis was performed on the CaP cell lines and immortal normal prostate epithelial cell lines p69 and RWPE1. The results showed that EphA6 protein expression was not detectable in prostate epithelial cells p69 and RWPE1 (Fig. 1C and 1D). However, EphA6 was detected in all the CaP cell lines and the expression was increased in metastatic derivative CaP cells (Fig. 1C and 1D). This interesting finding supports a potential role of EphA6 in CaP metastasis.

To investigate whether the results observed in CaP cell lines also hold true in clinical samples, we assessed EphA6 protein expression in 25 pairs of primary CaP tumor tissues and matched adjacent non-tumor tissues by immunohistochemistry. Minimal EphA6 protein was detected in the adjacent non-tumor tissues (Fig. 1E). In contrast, EphA6 protein was strongly expressed in primary CaP tumor tissues (Fig. 1E). The number of cells positive for EphA6 was significantly higher in the primary cancer tissues than in matched adjacent non-tumor tissues (Fig. 1F). These findings strongly indicate that EphA6 is indeed associated with CaP progression.

### Knock-down of EphA6 leads to low metastatic potential

**Figure 2:**
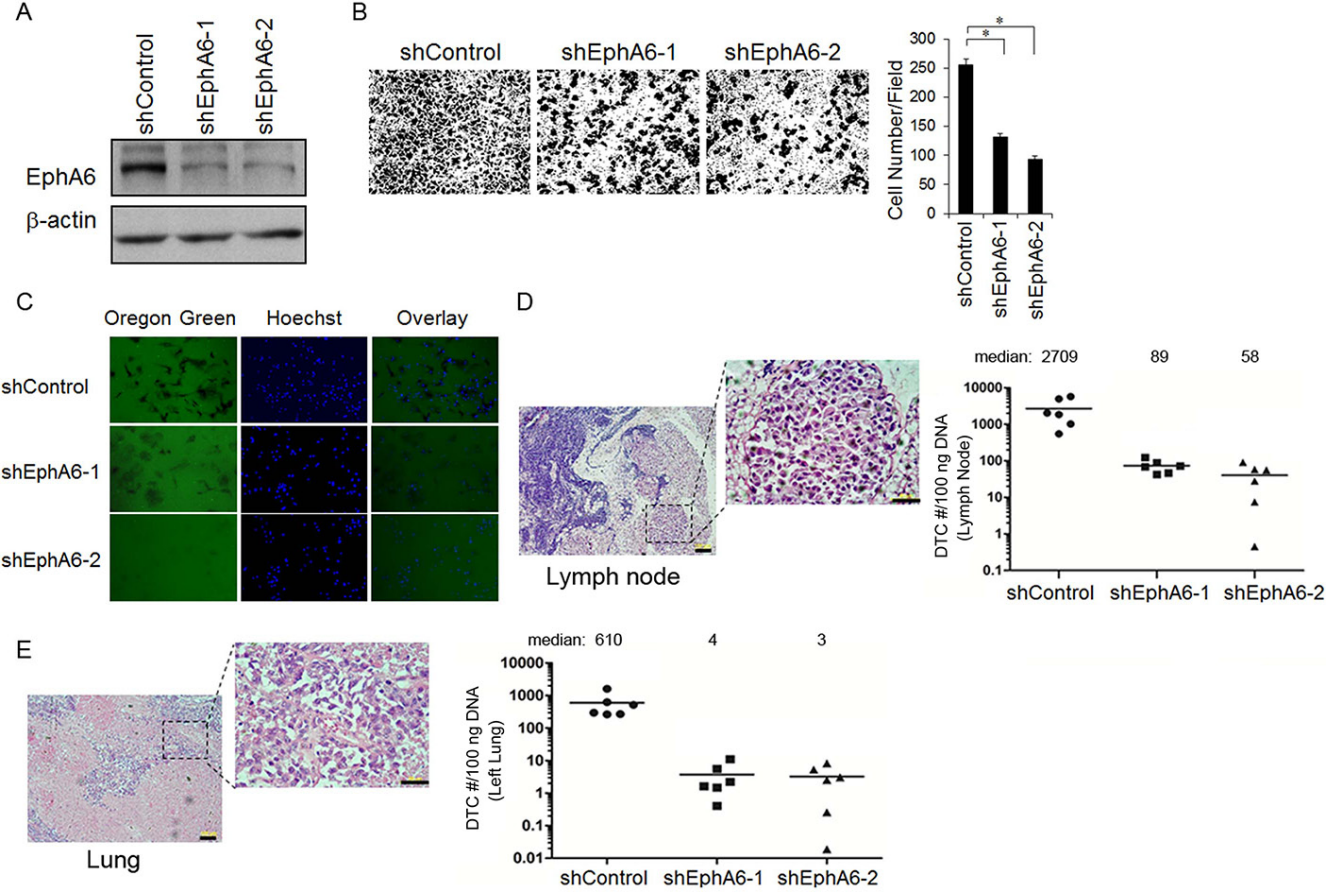
EphA6 knock-down in PC-3M CaP cells leads to low metastatic potential **A.** PC-3M cells were stably transduced with shRNA against EphA6 (vs. control vector), and EphA6 protein levels was assessed by Western blot analysis. **B.** Matrigel-based invasion assay was performed with PC-3M/shEphA6-1, PC-3M/shEphA6-2 or PC-3M/shControl cells using modified Boyden chambers with 10% FBS as a chemoattractant. Representative images were presented (Left panel). The cell numbers per field were counted and the results summarized in a bar graph (Right panel). *P < 0.05. **C.** The *in situ* invasiveness of PC-3M/shControl vs. PC-3M/shEphA6-1 or PC-3M/shEphA6-2 was assessed by *in situ* zymography using Oregon Green 488-labeled gelatin. The nuclei were labeled by hoechst. **D-E.** Subcutaneous xenograft CaP models were generated using PC-3M/shEphA6-1, PC-3M/shEphA6-2 or PC-3M/shControl cells. Tumors were harvested and the existence of metastatic tumor cells in local draining lymph nodes (LN) and lungs were assessed by qPCR-based detection of human Alu sequences. Representative images of the metastatic LN (D) and lung lesions (E) from a PC-3M/shEphA6-1 tumor stained with H&E were presented (Left panel). The quantification results were summarized in bar graphs (Right panel). Bars, 100 μm.

To determine a potential role of EphA6 in CaP metastasis potential, we first examined whether knocking down EphA6 affects the invasiveness of CaP cells. Due to low invasive and poor metastatic ability, LNCaP cells are not suitable for *in vivo* investigation of invasion and metastasis. The highly metastatic CaP cell line PC-3M was stably transfected with one of the two shRNA clones against EphA6 (shEphA6-1 or shEphA6-2) or control shRNA. Reduced EphA6 expression was confirmed by Western blotting results in both shEphA6-1 and shEphA6-2 transfected cell lines (Fig. 2A). Knock-down of EphA6 by shRNA resulted in 2- to 4-fold decrease in PC-3M invasiveness, as assessed by Boyden chamber-mediated invasion assay (Fig. 2B). Further, we analyzed the ability of PC-3M cells with EphA6 knock-down to degrade matrix *in situ* by Oregon Green 488-labeled gelatin-based *in situ* zymography assasy. shEphA6-transfected PC-3M cells had a decreased ability to locally digest the gelatin extracellular matrix compared with cells transfected with control shRNA (Fig. 2C).

We then tested whether EphA6 knock-down could inhibit spontaneous metastasis *in vivo* in a subcutaneous xenograft model using PC-3M/shControl, PC-3M/shEphA6-1, or PC-3M/shEphA6-2 cells in nude mice. When the primary tumors grown in the control mice exceeded 2 cm^3^ or ulcerated the skin (~ 49 days), the mice were sacrificed and the related organs were harvested and analyzed for human Alu sequence by qPCR for metastatic tumor cells. Samples harvested from PC-3M/shEphA6 groups presented with significantly decreased incidence of metastases to local draining lymph nodes (LN) (Fig. 2D) and lungs compared to the control group (*P* < 0.01) (Fig. 2E). These observations indicate that EphA6 may promote CaP metastasis.

### Knock-down of EphA6 decreases tumor angiogenesis

**Figure 3:**
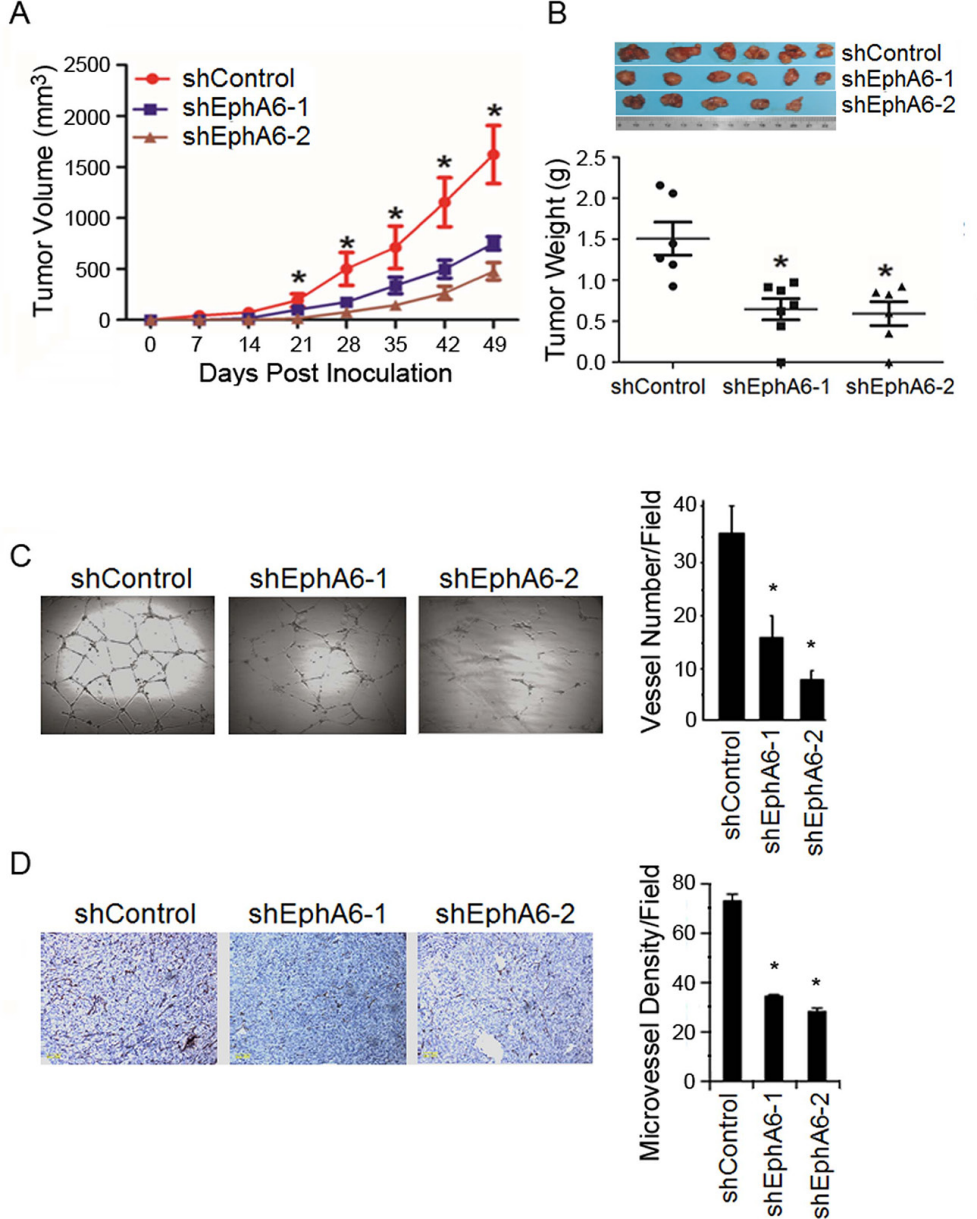
Knock-down of EphA6 decreases tumor angiogenesis **A.** Subcutaneous xenograft CaP models were generated using PC-3M/shEphA6-1, PC-3M/shEphA6-2 or PC-3M/shControl cells. Tumor growth was monitored over 7 weeks and the average tumor volumes of primary tumors were calculated. *P < 0.05. **B.** Tumors were removed and the weights measured. The middle horizontal lines represent the means in the groups (6 mice/group). *P < 0.05. **C.** *left*, Human endothelial cell tube formation. Human microvessel endothelial cells were plated onto Matrigel in the presence of 30% conditioned media from PC-3M/shEphA6-1, PC-3M/shEphA6-2, or PC-3M/shControl cells for 24 hours. *Right*, the mean numbers of tubes formed per visual field (five fields/group) from three independent experiments were presented. Bars, SE. *P < 0.001. **D.** CD34 staining of the primary s.c. PC-3M/shEphA6-1, PC-3M/shEphA6-2, or PC-3M/shControl tumors 49 days following implantation. Bars, 100 μm. Right panel, the quantification of the microvessel density was presented in a bar graph. *P < 0.001.

Reducing EphA6 by shRNA had no obvious effect on PC-3M cell proliferation *in vitro* (data no shown). Interestingly, we found that the primary tumors of PC-3M/shEphA6-1 and PC-3M/shEphA6-2 grew slower than the PC-3M/shControl tumors (Fig. 3A). The average tumor weights of the PC-3M/shEphA6-1 and PC-3M/shEphA6-2 primary tumors were significantly less than the control tumors (*P* < 0.05) (Fig. 3B). These intriguing results suggest that EphA6 may affect cells in the tumor environments rather than the proliferation of the tumor cells.

Tumor growth to a certain size relies on the development of blood vessels, which also provide an important route for metastatic dissemination. Considering that the Eph family has been reported to be involved in angiogenesis [4], we next investigated whether the suppression of tumor growth and metastasis by knock-down of EphA6 was due to decreased angiogenesis, at least partially. *In vitro* tube formation assay showed that conditioned media from PC-3M/shEphA6-1 or PC-3M/shEphA6-2 decreased the ability of human microvascular endothelial cells to form capillary-like tube structures in Matrigel in comparison with endothelial cells cultured in conditioned media from control cells (Fig. 3C). Further, EphA6 knock-down decreased the microvascular density (MVD), an indicator of angiogenesis, in PC-3M/shEphA6-1 or PC-3M/shEphA6-2 tumor tissues compared with PC3-M/shControl (Fig. 3D). Collectively, these data indicate that knock-down of EphA6 may contribute to the reduced tumor growth and metastasis through the inhibition of tumor neovascularization.

### Analysis of EphA6-regulated pro-metastatic genes

**Figure 4:**
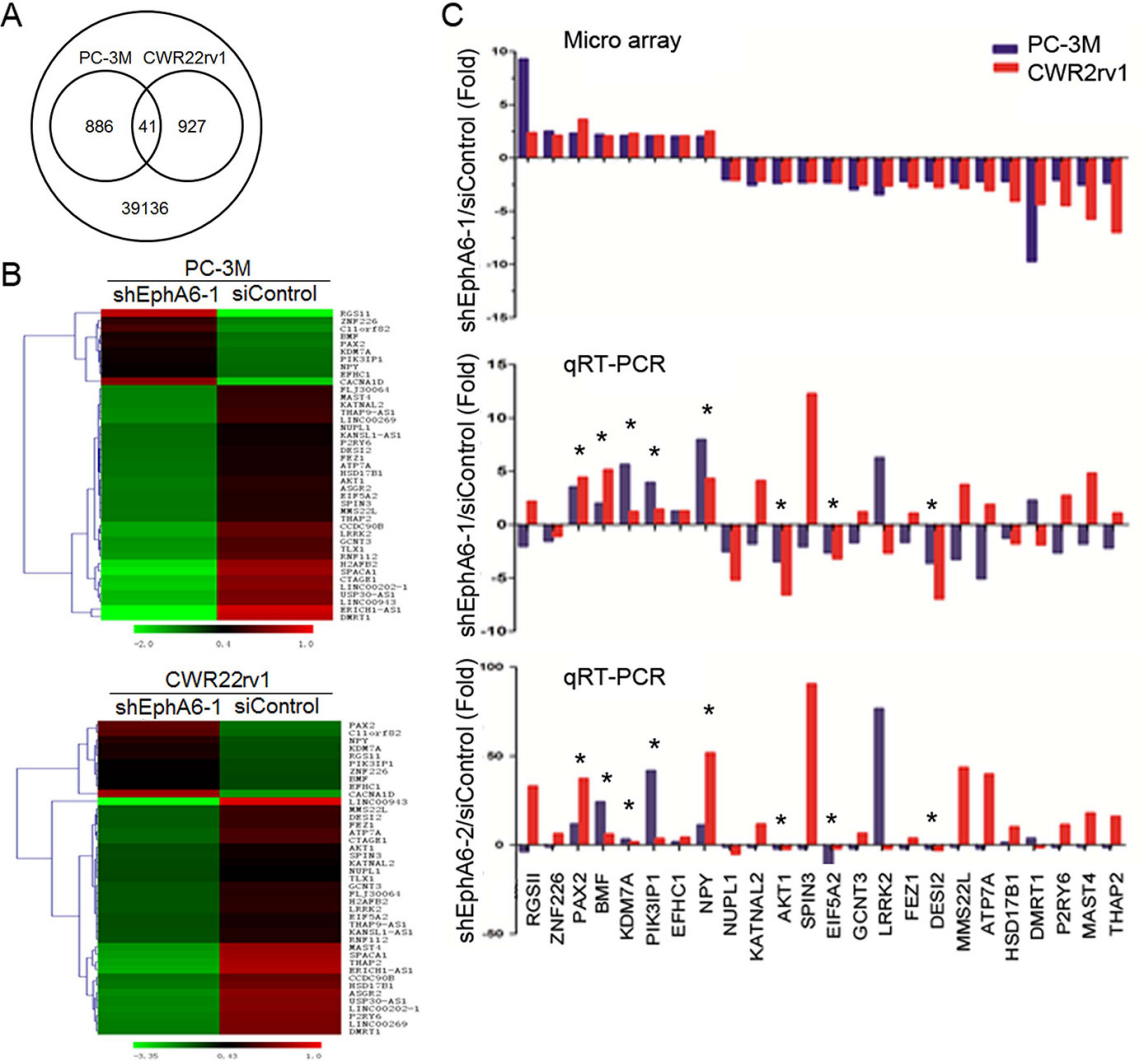
Identification of candidate pro-metastatic genes regulated by EphA6 **A.** Human whole-genome gene expression arrays were performed with DNAs isolated from PC-3M and CWR22rv1 cells transduced with shEphA6-1 or shControl. The Venn diagram shows unique and common genes differentially expressed (fold change > 1.5, *P* < 0.05) after EphA6 knock-down in PC-3M and CWR22rv1 cells. **B.** Heat maps of 41 commonly regulated genes after EphA6 knock-down in PC-3M and CWR22rv1 cells. **C.** The 24 genes related to metastasis were validated by qRT-PCR in PC-3M and CWR22rv1 cells. Their microarray results were presented on the top panel. *P < 0.05.

To examine whether decreased metastasis and angiogenesis after knock-down of EphA6 involves the regulation of gene expression, human whole-genome gene expression array was performed on PC-3M/shControl or CWR22rv1/shControl vs. PC-3M/shEphA6-1 or CWR22rv1/shEphA6-1 cells. Analysis of the array results identified 1854 genes with their expression changed ≥ 1.5-fold in shEphA6-1 transfected cells (Fig. 4A). Among these genes, 41 were common in both cell lines (Fig. 4A). The heat-maps of the 41 genes were presented in Fig. 4B. A pathway analysis revealed that among these 41 genes, 24 genes (8 up-regulated, 16 down-regulated) were involved in tumor or metastasis-associated processes. These processes include cell motility, invasiveness, angiogenesis and cell survival. The expression of the 24 genes was validated by qRT-PCR in both PC-3M and CWR22rv1 cell lines (Fig. 4C). Of these 24 genes, 8 genes including PAX2, PIK3IP1, BMF, KDM7A, NPY, AKT1, EIF5A2, and DESI2 were validated by qRT-PCR to be consistently regulated after EphA6 knock-down in both cell lines. Our results suggest that EphA6 contributes to the regulation of multiple genes involved in metastasis.

### EphA6 expression is positively correlated with PSA, vascular invasion and neural invasion of CaP cases

**Figure 5:**
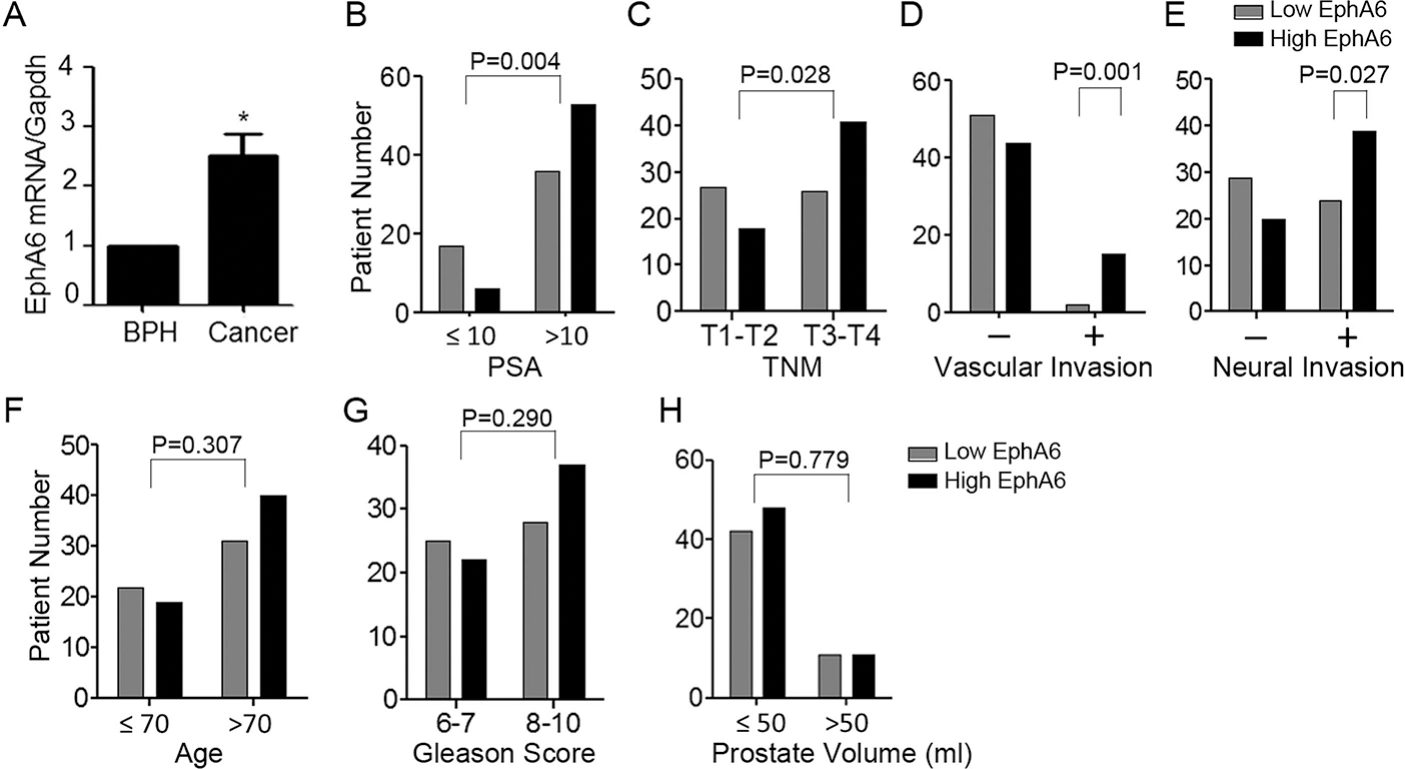
EphA6 expression is positively correlated with vascular invasion and neural invasion of CaP **A.** The expression of EphA6 mRNA in a cohort of 112 CaP tumor tissue samples and 58 benign prostate tissue samples from BPH patients was assessed by qRT-PCR. **B-H.** Correlation of EphA6 expression with clinical and histological parameters in prostate cancer patients: PSA (B), TNM (C), vascular invasion (D), neural invasion (E), age (F), Gleason score (G), and prostate volume (H)

To further investigate the relevance of EphA6 in CaP progression, we investigated the correlation between the expression of EphA6 mRNA and clinicopathological parameters in 112 CaP cases and 58 BPH cases by qRT-PCR. EphA6 mRNA expression levels in CaP tumor tissues were significantly higher than in the benign prostate tissues from the BPH patients (*P* < 0.001) (Fig. 5A). We then divided the 112 prostate cancer cases into 2 groups based on the EphA6 mRNA expression levels in tumor tissues. Patients with EphA6 expression below the average 2^−ddCt^ value of 2 were assigned to the low-EphA6 group (n = 53), and the rest cases were assigned to the high-EphA6 group (2^−ddCt^ >= 2, n = 59). Clinicopathological factors were then analyzed in relation to EphA6 levels. Significant correlations were identified between EphA6 expression and PSA level (*P* = 0.004, Fig. 5B), TNM staging (*P* = 0.028, Fig. 5C), vascular invasion (*P* = 0.001, Fig. 5D), and neural invasion (*P* = 0.027, Fig. 5E). However, we did not identify a correlation between EphA6 expression and age (*P* = 0.307, Fig. 5F), Gleason score (*P* = 0.290, Fig. 5G), or prostate volume (*P* = 0.779, Fig. 5H). These data strongly support a role of EphA6 in CaP progression and metastasis.

## DISCUSSION

Eph receptors have important roles in a variety of biological functions and also contribute to cancer development and progression. The current study identifies EphA6 as a potentially novel pro-metastatic gene amongst the Eph family in CaP. We observed that EphA6 is consistently overexpressed in CaP lymph node metastatic cell lines. EphA6 expression is high in CaP tumor tissues while almost undetectable in the adjacent non-tumor tissues. Furthermore, increased EphA6 expression is detected in 112 CaP tumor tissue samples compared with 58 benign prostate tissues from BPH patients. These data indicate that high EphA6 level is associated with CaP aggressiveness. Our finding is supported by a previous report showing that EphA6 is one of the up-regulated genes in the rapid-growing orthotopic castrated tumors derived from the androgen-independent CaP cell line LNCaP-19 [12]. We also observed that EphA6 tumor expression is positively correlated with vascular invasion, neural invasion, PSA and TNM staging in the 112 human CaP cases. However, there is no significant correlation between EphA6 expression in tumors and the Gleason scores. A possible explanation is that the Gleason score of samples start from 6, which represents already highly aggressive CaP. These data corroborates the correlation of EphA6 with CaP progression.

Metastasis requires a complex set of processes including specialized parameters of cell motility such as chemotaxis, adhesion, and invasion [13]. While employing shRNA to knock down EphA6, our study shows that decreased EphA6 can attenuate invasiveness and the degradation of extracellular matrix in PC-3M cells using *in vitro* assays and inhibit lung and lymph node metastasis *in vivo* in subcutaneous xenograft mouse models. These findings suggest that the promotion of these motility and invasion parameters contributes to the pro-metastatic activity of EphA6.

Experimental and clinical data show that the metastatic progression of CaP depends on tumor-associated angiogenesis [14]. Increasing evidence demonstrates an association between Eph/ephrin and neovascularization [15]. A recent microarray analysis on the gene expression profiles of Eph receptors demonstrates that EphA6 mRNA level is higher in adult human coronary artery endothelial cells than in adult human peripheral blood monocytes [16]. In addition, EphA6 mRNA is down-regulated in the myocardium of adult mouse after myocardial infarction [17]. Our study indicates that knock-down of EphA6 slows primary tumor growths, but did not affect cell proliferation rates *in vitro*, suggesting that knock-down of EphA6 mediated decrease in tumor growth is not due to changes in the proliferation rates of tumor cells. Therefore, we speculate that the potential roles of EphA6 in CaP development and metastasis may also involve angiogenesis. Our study shows that conditioned media generated from knock-down of EphA6 cells decreases endothelial cell tube formation *in vitro*. Further, CaP tumors with EphA6 knock-down present reduced angiogenesis *in vivo*. Taken together, these observations suggest that EphA6 may promote CaP progression and metastasis through enhancing angiogenesis, at least in part.

Phosphatidylinositol-3-kinase (PI3K)/AKT pathway is a major contributor to CaP progression [18, 19]. It is noteworthy that genome-wide gene expression analysis indicates that some genes regulated by EphA6 are either the components of PI3K/AKT pathway or associated with this pathway. For instance, knock-down of EphA6 decreased AKT, a major component of PI3K/AKT pathway. Our studies identified 24 metastasis-related genes that are consistently regulated in both PC-3M and CWR22rv1 cells by the knock-down of EphA6. Among the 24 genes, 8 genes were validated by qRT-PCR, and several of them are the targets of PI3K/AKT pathway. EIF5A2, an AKT target gene which promotes melanoma cell invasion, has decreased expression following EphA6 knock-down [20]. Moreover, the expression of PIK3IP1, a negative regulator of PI3K and suppressor of tumor development [21], increases after EphA6 knock-down. These findings suggest that EphA6 may play its role through an interaction with PI3K/AKT signaling pathway.

In conclusion, we demonstrate that EphA6 over-expression is associated with aggressive CaP and metastasis. Although further mechanistic investigation into the regulation of metastasis by EphA6 is necessary, the observed pro-metastatic activity of EphA6 in CaP supports EphA6 to be a potential target for cancer metastasis therapy.

## MATERIALS AND METHODS

### Cell culture

LNCaP, PC-3, CWR22rv1, RWPE-1 were purchased from Chinese Academy of cell bank. P69, RWPE-1, LNCaP, LNCaP/LN3, and CWR22rv1 cells were cultured in RPMI 1640 media supplemented with 10% FBS and incubated at 37°C in a humidified incubator containing 5% CO2. PC-3, PC-3M and PC-3M/LN4 cells were cultured in DMEM/F12 media supplemented with 10% FBS.

### Clinical samples

A total of 112 CaP tissue and 58 prostate tissue samples from BPH patients were obtained by needle biopsy between March and September 2013 from the Department of Urology in Huashan Hospital and the Department of Urology in Cancer Hospital affiliated with Fudan University (Shanghai, China). Written consents were obtained from all subjects prior to the recruitment. The study protocol was approved by the Ethics Committee of Huashan Hospital and Cancer Hospital of Fudan University. All tumor samples were confirmed to contain more than 80% tumor cells by histological examination of sequential sections by pathologists. Staging was assessed after pathological examination of formalin fixed specimens according to the 1997 TNM classification. The clinical and pathologic characteristics of the subjects are listed in Supplemental Table S1.

### Quantitative reverse transcriptase PCR (qRT-PCR)

Total RNA (1 μg) was isolated using Trizol reagent and subjected to reverse transcription reactions with random hexamer primers using the High Capacity cDNA Reverse Transcription kit (Applied Biosystems, Foster City, CA). qRT-PCR was performed on a 7900HT Sequence Detection system (Applied Biosystems) using FastStart Universal SYBR Green Master kit (Roche Diagnostics, Indianapolis, IN) and TaqMan^®^ Universal Master Mix (Applied Biosystems) following the manufacturer's instructions. Taqman primers and probe sets for Ephs and Ephrin family (Supplemental Table S2) were purchased from Applied Biosystems. GAPDH was used as a housekeeping control gene. Each test was done in triple replication and the 2^−ΔΔCt^ method was used to calculate the expression of genes. The mRNA expression heat-map of Eph receptors and ephrin ligands was generated by matrix2png (http://chibi.ubc.ca/matrix2png/bin//matrix2png.cgi).

### shRNA transfection

Lentiviruses expressing EphA6 shRNA (shEphA6-1 or shEphA6-2) or control shRNA were used for RNA interference. Viruses were generated in HEK293T cells harboring pLP/VSVg packaging plasmid and either plko-control shRNA or plko-EphA6 shRNA plasmid (shEphA6-1: GCAGCAGGGTTTACAACAT, shEphA6-2: GCATCTGCAGTACAGGATAT). PC-3M and CWR22rv1 cells were grown in 24-well plates to 80–90% confluence overnight and then transfected with 25 nM of control or shEphA6 for 24 h using Lipofectamine^TM^ reagent (Invitrogen) following the manufacturer's protocol. Following transfection, the cells were harvested and EphA6 knocknock-downown was confirmed by Western blot analysis.

### Subcutaneous xenograft tumor model

Male BALB/c nude mice (4–6 weeks, 18–20 g) were purchased from Slaccas Company (Shanghai, China) and maintained under specific pathogen-free (SPF) conditions. A total of 1 × 10^7^ PC-3M cells harboring shEphA6-1, shEphA6-2 or shControl in 0.1 ml saline suspension were mixed with 0.1 ml of Matrigel (BD, Heidelberg, Germany) and subcutaneously injected between the scapulae of narcotized (O_2_/CO_2_) mice (6 mice/group). The mice were assessed daily and weighed once a week. The tumor sizes were measured in a blinded manner with a dial caliper and tumor volumes calculated using the following formula: volume = width^2^ × length × 0.52. All animal protocols were approved by the Shanghai Medical Experimental Animal Care Commission.

When primary tumors of control mice exceeded 2 cm^3^ or ulcerated the skin (~ 49 days), mice were terminally narcotized and sacrificed. Primary tumors were removed, weighed and processed for histologic examinations. The right lungs and kidneys, and half of the hearts, spleens, livers, and lymph nodes were collected and fixed in formalin. The left lungs and kidneys, and the other half of the hearts, spleens, livers, and lymph nodes were homogenized in a sample disruptor (Tiangen, China) and subjected to DNA-extraction (TIANamp Genomic DNA Kit, Tiangen, China).

### Evaluation of human disseminated tumor cells (DTC) metastasis in the lymph nodes and lungs by PCR-based detection of human Alu sequences

To determine human DTC in PC-3M-inoculated mice, after terminal anesthesia, qPCR and melting curve analyses were performed on the lymph nodes and lungs with established primers specific for human Alu sequences as previously described [22]. DNA concentrations of all samples were quantified using NanoDrop1000 spectrophotometer (Thermo Scientific, USA) and normalized using AE buffer (Qiagen). qPCR was performed under the following conditions: 95°C for 10 min, 40 cycles at 95°C for 5 s, 65°C for 5 s, and 72°C for 20 s. Numerical data were determined against a standard curve established using murine blood DNA containing log-fold dilutions of DNA from 1 × 10^6^ PC-3M cells grown in culture. Negative controls were generated for each tissue type by testing DNA from un-inoculated mice of similar sex and age. qRCR assays were performed in duplicates. The independent experiments were repeated at least three times.

### Evaluation of tumor angiogenesis

The immunohistochemical staining of CD34 was used for microvascular density (MVD) counting as described previously [23]. Briefly, the areas containing large number of microvessels or “hot spots” with immunoreactivity against CD34 were identified at a low magnification (× 100). Five hot spots selected randomly were observed and the number of MVD was counted manually at × 200. Any brown-stained endothelial cells and their clusters that were clearly separated from the adjacent microvessels, tumor cells, and other connective tissues were considered as separate countable vessels. The mean score of the five hot spots was set as the level of MVD of each animal.

### Invasion assay

Modified Boyden chamber assays were performed as previously described [24]. Briefly, 5 × 10^4^ PC-3M cells harboring shEphA6-1, shEphA6-2 or shControl in 100 μl of serum-free medium were added to the wells of Matrigel^TM^ invasion chamber (BD). The lower chambers contained 10% FBS as a chemoattractant. The cells were allowed to migrate and invade for 24 h, and those that had not penetrated the membranes were wiped off with cotton swabs. Chamber membranes were fixed, stained with crystal violet and examined under a bright-field microscope. Values for migration and invasion were obtained by counting 6 fields per membrane (× 200) and presented as the average of three independent experiments.

### *In situ* zymography

The *in situ* zymography assays were performed as previously described [25]. Briefly, glass coverslips were coated with 0.2 mg/ml Oregon Green^®^ 488 dye-conjugated gelatin (molecular probes, G-13186), cross-linked in 0.5% glutaraldehyde, then incubated with 5 mg/ml NaBH4. The coverslips were sterilized with 70% ethanol and incubated in PBS. PC-3M cells infected with shEphA6-1, shEphA6-2 or shControl lentiviruses were plated on the coated coverslips, incubated at 37°C for 36 h. Cells were fixed and stained by Hoechst and examined by fluorescence microscopy.

### Tube formation assay

As previously described [26], conditioned medium was obtained by incubating PC-3M/control or PC-3M/shEphA6 cells for 24 h in medium containing 1% FBS. Human microvessel endothelial cells seeded in triplicate on a layer of previously polymerized Matrigel in 96 wells were incubated overnight with different concentrations of conditioned media and then photographed under a phase-contrast microscope.

### Gene expression array

Total RNA was isolated using Trizol from four samples: PC-3M/shEphA6-1, PC3M/shControl, CWR22rv1/shEphA6-1, and CWR22rv1/shControl cell line. RNA were quantified using NanoDrop-1000 spectrophotometer and evaluated for degradation using Agilent Bioanalyzer 2100 (Agilent Technologies, Santa Clara, CA). Samples with RNA Integrity Value (RIN) > 7 were processed for gene expression array analysis using Agilent Human whole-genome gene expression array (Agilent Technologies, Santa Clara, CA). Total RNA was amplified and labeled by Low Input Quick Amp Labeling Kit (Agilent technologies), following the manufacturer's instructions. Labeled cRNA were purified by RNeasy mini kit (QIAGEN, Germany), and then mixed with hybridization reagents and hybridized overnight at 58°C to the Chips. Following washing and staining with Cy3-labeled cRNA, the Chips were imaged using Agilent Microarray Scanner (Agilent technologies) to measure fluorescence intensity at each probe. The intensity of the signal corresponds to the quantity of the respective mRNA in the original sample. Data were extracted with Feature Extraction software 10.7 (Agilent technologies). Raw data were normalized by Quantile algorithm, Gene Spring Software 11.0 (Agilent technologies). qRT-PCR was used to validate the array results. Our raw data has been deposited in NCBI's Gene Expression Omnibus (GEO: http://www.ncbi.nlm.nih.gov/geo/) and are accessible through GEO series accession number GSE66335. The sequences of the primers were designed using Primer-BLAST (Supplemental Table S3).

### Immunohistochemistry (IHC) and imaging

Twenty-five pairs of primary tumor tissues and matched adjacent non-tumor tissues were obtained from patients after prostatectomy and fixed in 10% buffered formalin, embedded in paraffin and sectioned at 4 microns. Slides were de-parafinized in several baths of xylene and then rehydrated in graded alcohol series followed by ddH_2_O. Slides were incubated in 1 × pH 6 citrate buffer (Maixin) for 10 min and then in 3% H_2_O_2_ for 15 min. To block non-specific binding, tissues were incubated with normal goat serum for 1 h. Anti-EphA6 polyclonal antibody (dilution 1:100, Abgent, CA, US) was diluted in 1% BSA and incubated 1 h at 37°C, followed by staining using HRP-conjugated secondary antibody (Dako, UK) for 1 h at room temperature. To reveal endogenous peroxidase activity, slides were incubated with DAB substrate and stained with hematoxylin. Slides were scanned with Olympus BX53 microscope and viewed using Cellsens Entry (Olympus). The intensity of immunoreactivity (intensity score) was judged on an arbitrary scale of 0–4: 0, negative; 1, weak; 2, moderate; 3, strong and 4, very strong. Additionally, samples were given a percentage score based on the percentage of tissue displaying each intensity score. Each score was represented as a histoscore in which the percentage score was multiplied by the intensity score, giving a range of 0 to 400.

### Statistics

Statistical significances between groups were determined by two-tailed student's *t*-test. The differences in gene expression levels between CaP tumor tissues and BPH tissue specimens were tested by chi-square test. All statistical analyses were performed by using SPSS 16.0 software program. *P* < 0.05 was considered statistically significant.

## SUPPLEMENTARY TABLES



## Abbreviations

CaP: prostate cancer
Ephs: Eph receptors
BPH: benign prostate hyperplasia
qRT-PCR: real-time quantitative RT-PCR
LN: lymph nodes
MVD: microvascular density
PI3K: Phosphatidylinositol-3-kinase
DTC: disseminated tumor cells
RIN: RNA Integrity Value
IHC: Immunohistochemistry
