# Supplementary material for: EphA6 promotes angiogenesis and prostate cancer metastasis and is associated with human prostate cancer progression
Source: Oncotarget. 2015 May 27;6(26):22587–97. doi: 10.18632/oncotarget.4088 (PMC4673184; doi:10.18632/oncotarget.4088)
Supplement: Supplementary file 1 [file oncotarget-06-22587-s001.pdf]

## SUPPLEMENTARY TABLES

**Supplementary Table S1. Clinical and histological characteristics of patients enrolled in the study**

| | Prostate cancer (%) | BPH (%) |
|---|---|---|
| Case, *n* | 112 | 58 |
| Age (mean ± SD, years) | 68.2 ± 9.5 | 65.2 ± 10.7 |
| TNM | | |
| T1 | 2(1.8) | |
| T2 | 43(38.4) | |
| T3 | 31(27.7) | |
| T4 | 36(32.1) | |
| Gleason score | | |
| 6–7 | 47(42.0) | |
| 8–10 | 65(58.0) | |
| PSA (ng/ml) | | |
| < 4.0 | 8(7.1) | 11(18.9) |
| 4.0–10.0 | 15(13.4) | 24(41.4) |
| > 10.0 | 89(79.5) | 23(39.7) |
| Prostate volume(ml) | | |
| < 30 | 43(38.4) | 17(29.3) |
| 30–50 | 47(42.0) | 18(31.0) |
| > 50 | 22(19.6) | 23(39.7) |

## Supplementary Table S2. EPH probe information

| Assay ID | Gene Symbol | RefSeq | Amplicon Length (bp) |
|---|---|---|---|
| Hs00178313_m1 | EPHA1, hCG20689 | NM_005232.4 | 66 |
| Hs00543478_m1 | EPHA10 | NM_173641.2 | 125 |
| Hs00171656_m1 | EPHA2, hCG24712 | NM_004431.3 | 55 |
| Hs00739096_m1 | EPHA3, hCG33008 | NM_182644.2 | 101 |
| Hs00177874_m1 | EPHA4, hCG1811889 | NM_004438.3 | 91 |
| Hs00300724_m1 | EPHA5, hCG1810817 | NM_004439.5 | 72 |
| Hs00297133_m1 | EPHA6, hCG1811380 | NM_173655.2 | 95 |
| Hs00177891_m1 | EPHA7, hCG32399 | NM_004440.3 | 88 |
| Hs01025610_m1 | EPHA8, hCG39973 | NM_001006943.1 | 128 |
| Hs01057849_m1 | EPHB1, hCG1642595 | NM_004441.4 | 59 |
| Hs00362096_m1 | EPHB2, hCG1812037 | NM_017449.3 | 67 |
| Hs00177903_m1 | EPHB3, hCG16839 | NM_004443.3 | 80 |
| Hs00174752_m1 | EPHB4, hCG20448 | NM_004444.4 | 82 |
| Hs01071144_m1 | EPHB6, hCG20681 | NM_004445.3 | 58 |
| Hs00358886_m1 | EFNA1, hCG1745153 | NM_182685.1 | 67 |
| Hs01023290_m1 | EFNA2, hCG2039548 | NM_001405.3 | 83 |
| Hs00191913_m1 | EFNA3, hCG20022 | NM_004952.4 | 98 |
| Hs00193299_m1 | EFNA4, hCG20023 | NM_182689.1 | 118 |
| Hs00157342_m1 | EFNA5, hCG1736717 | NM_001962.2 | 98 |
| Hs00270004_m1 | EFNB1, hCG15073 | NM_004429.4 | 63 |
| Hs00187950_m1 | EFNB2, hCG27196 | NM_004093.3 | 63 |
| Hs00154861_m1 | EFNB3, hCG42021 | NM_001406.3 | 74 |
| Hs99999905_m1 | GAPDH, hCG2005673 | NM_002046.3 | 122 |

**Supplementary Table S3. Primers used in the Taqman qRT-PCR**

| Gene | Primer | ProductSize (bp) |
|---|---|---|
| PIK3IP1 | Forward: TGGCATCATCTTGGGCTACTC | 120 |
| | Reverse: GGGTTGGTGAAGGCAGACAA | |
| PAX2 | Forward: TGCAGTTGGTCCCTCATCCT | 83 |
| | Reverse: CTCCAGTGGGTTGCACACAA | |
| RGS11 | Forward:CAGCAGCAAAACCCTTTGTTC | 105 |
| | Reverse: AGGTTCTAGGTGCATCTTTCCAG | |
| BMF | Forward: TCAGTGCATTGCAGACCAGT | 78 |
| | Reverse: ATCTGCCACCACACACGATT | |
| KDM7A | Forward: TGCTGTCTATGGCAGGGTTG | 71 |
| | Reverse: ACCACTGCAGTCTGTGCTTT | |
| NPY | Forward: GGAAAACGATCCAGCCCAGA | 91 |
| | Reverse: CAGGGTCTTCAAGCCGAGTT | |
| EFHC1 | Forward: TCTTCCGGGCACGTCCTTTA | 215 |
| | Reverse: CAGGTGGGGCTTGTTTAGGT | |
| ZNF226 | Forward: GGCTTCTGTTACAGCCCAGT | 110 |
| | Reverse: GTCTGTAGATGAGCGCCCTG | |
| AKT1 | Forward: TCTTTGCCGGTATCGTGTGG | 143 |
| | Reverse: TTGGTCAGGTGGTGTGATGG | |
| NUPL1 | Forward: GCGTCTAGCAACCCTTCTGT | 194 |
| | Reverse: GGCGTTCCCAGAGTTAATCC | |
| KATNAL2 | Forward: GAAGCCTGCGGATGAAGACA | 109 |
| | Reverse: ACCAGCAGCATGAAAATGGAC | |
| SPIN3 | Forward: ACCGTTCTGGATCAGCTCCT | 159 |
| | Reverse: CACACCGGTGGACACATTAC | |
| EIF5A2 | Forward: GCATGGTCATGCCAAGGTTC | 169 |
| | Reverse: ACCAGTTTCTGTCAGCAGGG | |
| GCNT3 | Forward: TCAAAGAGGCGGTCAAAGCA | 83 |
| | Reverse: GGAGGCATAAACCACCCGAA | |
| LRRK2 | Forward: ATGATGACAGCACAGCTAGGA | 95 |
| | Reverse: ATCTCTTTCTGCTTTTGTGTACC | |
| FEZ1 | Forward: AGGAGGAGACCCTTCAGGAC | 195 |
| | Reverse: GAGGCTCCTCGTTGATACCG | |
| DESI2 | Forward: GTGGTGCTCAACGTGTACGA | 120 |
| | Reverse: AGGATGGCCACCATAAGCAA | |

(*Continued*)

| Gene | Primer | ProductSize (bp) |
|---|---|---|
| MMS22L | Forward: ACTGCTGCCACATGCAGTAT | 193 |
| | Reverse: AGCTGGAAGAAATCGCCCAA | |
| ATP7A | Forward: GGACCACCACTTTGCAACTC | 168 |
| | Reverse: TACCAGCCTCCGAAAAACTGT | |
| HSD17B1 | Forward: TGCTCATCACCGGCTGTTC | 94 |
| | Reverse: AACGTGGCATACACTTTGAAGC | |
| THAP2 | Forward: AGAATGGGTTCGCCTGGTTA | 114 |
| | Reverse: AGTCGTCGAGTTTGTCCTGTT | |
| MAST4 | Forward: CCAGTGCCTCAGCCCATTTT | 125 |
| | Reverse: GGAACAGGATGAAGAGACCGT | |
| P2RY6 | Forward: CCGCGAGAACTTCAAGCAAC | 180 |
| | Reverse: GGCAGGGAGCAGGCATATAG | |
| DMRT1 | Forward: CAGGAAACCAGTGGCAGATGA | 90 |
| | Reverse: AAGAGGGAGGCGGGTAGTAA | |
| GAPDH | Forward: GAGAAGGCTGGGGCTCATTT | 231 |
